# Potentiating pneumococcal glycoconjugate vaccine PCV13 with saponin adjuvant VSA-1

**DOI:** 10.3389/fimmu.2022.1079047

**Published:** 2022-12-12

**Authors:** Hyunjung Kim, Jigui Yu, Di Bai, Moon H. Nahm, Pengfei Wang

**Affiliations:** ^1^ Department of Chemistry, University of Alabama at Birmingham, Birmingham, AL, United States; ^2^ Department of Medicine, University of Alabama at Birmingham, Birmingham, AL, United States; ^3^ World Health Organization (WHO) Pneumococcal Serology Reference Laboratory, University of Alabama at Birmingham, Birmingham, AL, United States

**Keywords:** *Momordica* saponin, adjuvant, immunostimulant, pneumococcal vaccine, VSA-1

## Abstract

VSA-1 is a semisynthetic saponin adjuvant prepared from naturally occurring *Momordica* saponin and capable of stimulating antigen-specific humoral and cellular immune responses. Its immunostimulating activity in enhancing the immune responses induced by the clinical glycoconjugate pneumococcal vaccine PCV13 is compared with QS-21 in female BALB/c mice. Both VSA-1 and QS-21 boosted IgG and opsonic antibodies titers against seven selected serotypes, including serotypes 3, 14, and 19A that are involved in most PCV13 breakthroughs. Since VSA-1 is much more accessible and of lower toxicity than QS-21, it can be a practical saponin immunostimulant to be included in a new glycoconjugate pneumococcal vaccine formulation.

## Introduction

*Streptococcus pneumoniae* is a leading cause of bacterial pneumonia and meningitis, accounting for an estimated 660,000 lower respiratory tract infection-related deaths and 9,600 meningitis-related deaths in adults aged >50 years of age globally each year (1). Mortality rates are high especially in the very young, elderly, and immunocompromised individuals. Vaccines can be an effective way to prevent infections by *S. pneumoniae*, including drug-resistant strains. There are two types of clinical pneumococcal vaccines: pneumococcal polysaccharide vaccine (*e.g*., PPV23, composed of purified pneumococcal capsular polysaccharides (CPS) of 23 serotypes of *S. pneumoniae*) and pneumococcal glycoconjugate vaccine (*e.g*., PCV13, composed of purified CPS of 13 serotypes of *S. pneumoniae* individually conjugated to diphtheria toxin protein carrier CRM197) (2, 3). However, both vaccines have limitations (2–8), for example, PPV23 is not effective in children younger than 2 years old, and only 60-70% effective against invasive disease (9). The use of PCV13 substantially reduced invasive pneumococcal disease (IPD) caused by PCV13 vaccine serotypes in all age groups, but the reductions of IPD in each of the 13 vaccine serotypes of PCV13 varied among serotypes. PCV13’s effectiveness against serotype 3 was not significant (10), and most vaccine breakthroughs in children involve serotype 3 (4, 11–13), and there are also cases involving serotypes 14 and 19A (14–17). In addition, immunosenescence is a noticeable issue with current pneumococcal vaccines; PCV13 is 75% effective against IPD in adults older than 65 years. It is therefore desirable to improve the efficacy of glycoconjugate vaccines.

A viable way to potentiate humoral and cellular immune responses is to add an immunostimulating adjuvant to the vaccine (18). Adjuvants constitute an indispensable element of modern vaccines. They (a) enhance the ability of a vaccine to elicit strong and durable immune responses, especially in immunologically compromised individuals such as immunologically immature neonates, the aged, and immune suppressed individuals; (b) reduce antigen dose and the number of immunizations; and (c) modulate the nature of immune response (19). There are only a few adjuvants (e.g., alum, AS04, MF59, AS03, CpG, and AS01b) approved by the FDA for human use (20–24). PCV13 contains alum (various aluminum salts), the most used adjuvant; however, alum is a weak adjuvant and primarily enhances Th2 humoral immune responses without Th1 help.

QS-21 is a saponin adjuvant known for its capacity of inducing both Th1 and Th2 immune responses. It was recently approved as a component of adjuvant AS01b (25, 26) used in GlaxoSmithKline’s (GSK) shingles vaccine, Shingrix^®^, one of the most successful vaccine launches in recent years (25, 27). The protection offered by QS-21 vaccines is highly durable. QS-21 vaccines are effective for broad use across age groups: Shingrix^®^ is highly effective in older individuals (≥70 years) (28); and the GSK’s QS-21 containing malaria vaccine, MOSQUIRIX^®^, has been used to protect pediatric populations (29). However, QS-21 has its own limitations. It is a natural product isolated from the tree bark of *Quillaja saponaria* Molina (QS), an evergreen tree native to temperate central Chile. It has a severe supply issue; the current global supply of natural QS-21 may not be sufficient for widespread clinical use for various anti-infection vaccines (30, 31). Its limited supply, along with chemical instability, dose-limiting toxicity, and laborious and low-yielding purification, hinder its wider use (30, 31).

**Scheme 1:**
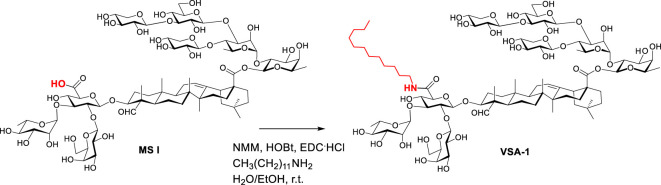
Preparation of VSA adjuvants from natural *Momordica* saponins.

In pursuit of practical alternatives to QS-21, Wang et al. discovered VSA-1 adjuvant based on extensive structure-activity-relationship studies (32–36). VSA-1 is a semisynthetic saponin which can be synthesized in only one-step from naturally occurring *Momordica* saponins (MS) isolated from the widely available and inexpensive seeds of *Momordica cochinchinensis* SPRENG (MC), a perennial vine (Synthesis of VSA-1 from MS I is depicted in **Scheme 1**) (34). VSA-1 can induce a strong antigen-specific, mixed Th1/Th2 immune response mirroring QS-21 and it is much less toxic than natural QS saponins (34). Recently, a split virus flu vaccine showed that VSA-1 has similar/superior adjuvant activity to QS-21 in terms of stimulating humoral and cellular immune responses. Thus, it has the potential to be an effective and inexpensive alternative to QS-21 for various high-volume vaccination needs, especially for anti-infection vaccines.

## Materials and methods

### Commercial vaccines

Each human dose of PCV13 (trade name Prevnar 13 by Pfizer) is available in 0.5 mL single-dose pre-filled syringes. It contains 2.2 μg of polysaccharide (PS) from each of 12 serotypes (*i.e*., 1, 3, 4, 5, 6A, 7F, 9V, 14, 19A, 19F, 18C, and 23F) and 4.4 μg of polysaccharide from serotype 6B conjugated to CRM197, along with 125 μg of alum adjuvant.

### Semisynthetic vaccine adjuvant

Synthesis of VSA-1: The published general procedure of synthesizing MS derivatives was used (34). Thus, MS I (120 mg, 0.07 mmol) in ethanol (3.0 mL) and water (1.0 mL) was added dodecylamine (50.0 mg, 0.27 mmol), *N*-methylmorpholine (NMM) (91.0 mg, 0.90 mmol), hydroxybenzotriazole (HOBt) (83.0 mg, 0.54 mmol), and 1-ethyl-3-(3-dimethylaminopropyl)carbodiimide hydrochloride (EDC.HCl) (107.0 mg, 0.54 mmol) at room temperature. The reaction mixture was stirred for 1 day, and was then filtered. The filtrate was directly purified with reverse-phase high performance liquid chromatography (RP HPLC) by using a Prep C18, 250x10 mm, 5-micron column, and H_2_O/acetonitrile (MeCN) gradients (90%-10% H_2_O over 45 minutes with a 3 mL/min flow rate). The product fraction was concentrated on a rotary evaporator at room temperature to remove MeCN, and the remaining water was then removed on a lyophilizer to provide the derivative as a white solid.

### Mice immunizations

BALB/c mice used in this study were purchased from the Jackson Laboratory and maintained within an environmentally controlled, pathogen-free animal facility at the University of Alabama at Birmingham (UAB). Each human dose of PCV13 (trade name Prevnar 13 by Pfizer) is available in 0.5 mL single-dose pre-filled syringes. QS-21 and VSA-1 were dissolved in sterile distilled water to give their respective stock solution at 1.0 mg/mL. Each mouse dose contained 50 μL of PCV13 plus 20 μL of QS-21 or 50 μL of VSA-1, diluted to a total volume of 200 μL with 0.9% Normal Saline. Groups of female BALB/c mice (8-10 weeks of age, six per group) were immunized *via* the subcutaneous route (*s.c*.) with 200 μL of saline, PCV13, PCV13 plus QS-21, or PCV13 plus VSA-1 (two sites/mouse at dorsal, 100 μL/site) on days 0, 14 and 28. Serum samples were collected prior to the first and the third immunizations and at 2 weeks following the last immunization. Equal volumes of the six sera in each group were pooled together to create serum pools for each group. The serum was obtained after centrifugation and stored at −20 °C until assayed. All studies were performed according to National Institutes of Health guidelines, and protocols were approved by the UAB Institutional Animal Care and Use Committee.

### ELISA

The World Health Organization (WHO)-approved ELISA assay described for human pneumococcal antibodies (37, 38) was adapted for mouse serum as described below. Briefly, each well of a 96-well microtiter plate was coated with 100 μL of PBS with a CPS at a pre-determined concentration. CPS of the seven serotypes (3, 4, 6B, 9V, 14, 19A, 19F, and 22F) were from American Type Culture Collection (ATCC). Plates were incubated at 37 °C for 5 h in a humidified chamber, except type 3 PS which was coated at room temperature for 2 hours. The coated plates were washed with Washing Buffer (TBS-0.1% Brij-35 solution) and blocked with PBS containing 0.5% BSA, 0.05% Tween 20 and 0.02% NaN_3_. To the PS-coated microtiter plates, was loaded 50 μL of the serum pool diluted as below. The serum pools were made by mixing equal volume of individual mouse serum in each group. The resulting serum pools were initially diluted 1:50 and then 3-fold serially diluted in Antibody Buffer (PBS with 0.1% BSA, 0.05% Tween-20 and 0.02% NaN_3_) with 5 μg/ml of teichoic acid (the Statens Serum Institute in Denmark) and 5 μg/ml 22F capsule (ATCC). The two absorbents were added to neutralized non-specific binding of irrelevant antibodies (38). The plates were incubated overnight at room temperature in a humidified box. After washing five times, 100 μL of diluted alkaline phosphatase-conjugated goat mouse immunoglobulin (Southern Biotech, Birmingham, AL) in Antibody Buffer was added to each well. After another 1-h incubation, the plates were washed five times, and 100 μL of the substrate solution containing *p*-nitrophenyl phosphate (Sigma) was added to each well. After a 1-h incubation at room temperature, the optical density (OD) was measured at 405 nm and at 690 nm. The detailed protocol of the WHO ELISA can be found at our website (http://www.vaccine.uab.edu) (38).

### Opsonophagocytosis assay

Opsonophagocytosis assay for serotypes 3, 4, 6B, 9V, 14, 19A, and 19F was performed using the 4-fold multiplexed opsonization assay (39). Briefly, 10 μL of bacterial suspension (∼0.5 × 10^5^ CFU/ml of each serotype) and 20 μL of serially diluted antiserum were incubated in a microtiter plate for 30 min at RT with shaking. Then 10 μL of 3- to 4-week-old rabbit serum as the complement source (PelFreeze Biologicals, Rogers, AK) and 40 μL of differentiated HL60 cells (4 × 10^5^ cells) were added to each well and the plates were incubated at 37°C in 5% CO_2_ with shaking for 45 min. An aliquot of the final reaction mixture (10 μL) was spotted onto four different Todd Hewitt Broth with yeast extract (THY) agar plates (39), and overlay agar containing one of the four antibiotics (optochin, spectinomycin, streptomycin, or trimethoprim) was applied to each THY agar plate. After an overnight incubation at 37 °C, the number of bacterial colonies was enumerated. Opsonic indices were determined as the interpolated serum dilution that kills 50% of bacteria. A detailed protocol can be found our website (http://www.vaccine.uab.edu).

## Results and discussion

**Figure 1:**
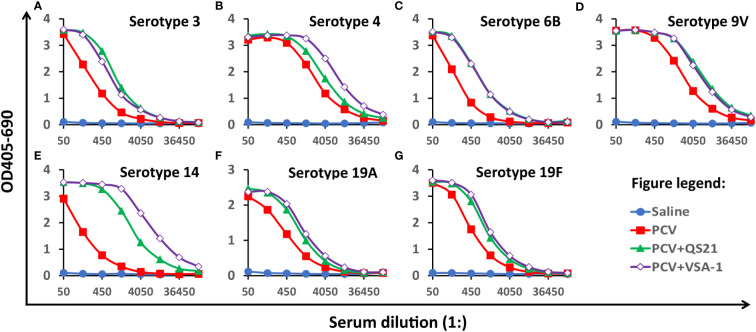
Serum antibody activity to seven serotypes on Day 42, **(A)** 3, **(B)** 4, **(C)** 6B, **(D)** 9V, **(E)**14, **(F)** 19A, and **(G)** 19F. BALB/c mice (8-10 weeks of age, six per group) were immunized *via* the subcutaneous route (*s.c*.) on days 0, 14 and 28. Serum samples were collected prior to the first immunization and at 2 weeks following the last immunization. The pooled serum samples of each group were analyzed by ELISA. The Y-axis shows the bound antibody (OD405-OD690) at various serum dilutions (X-axis). The serum pool from the mice immunized with saline had very small amounts of antibodies for all serotypes and its data were plotted close to the horizontal axis. Similarly, the data from pre-immune serum samples showed undetectable antibody for all serotypes (data not shown).

Herein we report our results of comparing VSA-1 and QS-21 in enhancing the immune responses induced by the clinical glycoconjugate pneumococcal vaccine PCV13. Each human dose of PCV13 (trade name Prevnar 13 by Pfizer) is available in 0.5 mL single-dose pre-filled syringes. It contains 2.2 μg of polysaccharide from each of 12 serotypes (*i.e*., 1, 3, 4, 5, 6A, 7F, 9V, 14, 19A, 19F, 18C, and 23F) and 4.4 μg of polysaccharide from serotype 6B conjugated to CRM197, along with 125 μg of alum adjuvant. Thus, groups of female BALB/c mice (8-10 weeks of age, six per group) were immunized *via* the subcutaneous route (*s.c*.) with saline (group A, negative control), PCV13 (group B), PCV13 plus QS-21 (20 µg) (group C), or PCV13 plus VSA-1 (100 µg) (group D) on days 0, 14 and 28. We used one tenth of one human dose of PCV13 for each mouse dose. Serum samples were collected prior to the first and the third immunizations and at 2 weeks following the third immunization. Equal volumes of the six sera in each group were pooled together to create serum pools for each group. ELISA was used to assess the antibody activity toward seven PCV13 serotypes, *i.e*., 3, 4, 6B, 9V, 14, 19A, and 19F (**Figure 1A–G**) (40).

The serum samples collected from all the four groups prior to the first immunization and the serum of saline control post the two or three immunizations (Days 28 and 42) showed no antigen-specific antibody titers (**Figure 1** and **Table 1**). PCV13 induced significant antibody responses to the seven tested serotypes on Days 28 and 42, and inclusion of a saponin adjuvant to PCV13 enhanced antigen-specific antibody responses to all seven serotypes compared with the PCV13 control group. When comparing the dilutions that gave OD of 6x background (i.e., antibody titers), VSA-1 and QS-21 showed similar adjuvant activities for serotypes 3, 6B, 9V, 19A, and 19F, but VSA-1 was superior to QS-21 for serotypes 4 and 14 on Day 42 (**Table 1**).

**Table 1:** ELISA Titer to Different Vaccine Serotypes^a^.

Sera			Serotype						
			3	4	6B	9V	14	19A	19F
Pre-immune			<50	<50	<50	<50	<50	<50	<50
Day-28	A (Saline)		<50	<50	<50	<50	<50	<50	<50
	B (PCV only)		2,465	6,306	63	16,186	153	1,003	1,334
	C (+QS21)		12,093	10,229	228	33,432	3,781	830	4,672
	D (+VSA1)		6,213	8,693	107	30,068	17,566	1,285	1,929
Day-42	A (Saline)		<50	<50	<50	<50	<50	<50	<50
	B (PCV only)		2,193	24,924	981	22,997	1,311	2,348	3,747
	C (+QS21)		9,243	53,797	5,794	91,990	24,828	4,693	8,853
	D (+VSA1)		8,764	>109,350	5,332	69,577	103,595	7,012	11,791

^a^Dilution that gives OD of 6x background.

The ELISA results provided evidence of antibody responses to the different vaccine formulations; however, these data do not indicate the ability of the antibodies to opsonize and kill bacteria, thus do not provide direct evidence of immune protection. Vaccine-induced immune protection against encapsulated *S. pneumoniae* is primarily mediated by opsonic antibodies that bind CPSs (41, 42). Opsonophagocytosis assay (OPA) is an important tool to evaluate the capacity of sera to kill the bacteria (40, 42, 43). OPA for serotypes 3, 4, 6B, 9V, 14, 19A, and 19F was performed using the 4-fold multiplexed opsonization assay for samples obtained on days 0 and 42 (39). Opsonic titers are defined as the reciprocal of the interpolated serum dilution that kills 50% of the bacteria. The OPA data show that inclusion of a saponin adjuvant in PCV13 enhance opsonic titers for all serotypes (**Table 2**). With QS-21, PCV13-induced opsonic titers increased in the range of 1.9-4.9 fold for serotypes 3, 4, 6B, 9V, 19A, and 19F, and a 14.9-fold increase for serotype 14. VSA-1 improved opsonic titers against serotype 14 even more, with an 18.2-fold increase, and a 2.1-4.1 fold increase for other serotypes except for serotypes 9V (x 0.8) (**Table 2**), even though it increased IgG response against 9V by 2.5 fold compared with PCV13 alone (**Table 1**). Comparison between VSA-1 and QS-21 shows that VSA-1 was superior to QS-21 in enhancing opsonic titers for serotypes 4, 6B, 14, and 19A, by 1.8, 2.0, 1.2, and 1.1 fold respectively. QS-21 was superior to VSA-1 for serotypes 3, 9V, and 19F, by 1.3, 2.4, and 1.2 fold, respectively.

**Table 2:** OPA Titer to Different Vaccine Serotypes^a^.

Sera	Serotypes						
	3	4	B	9V	14	19A	19F
Pre-immune	<20	<20	<20	<20	110	<20	<20
Post-A (Saline)	<20	<20	<20	<20	83	<20	<20
Post-B (PCV only)	673	7124	2511	4613	2398	1469	926
Post-C (+QS21)	3288	11298	5226	9235	35835	2779	2836
Post-D (+VSA1)	2514	19825	10296	3775	>43740^b^	3054	2343

^a^The opsonization titer was defined as the highest dilution of the serum that kills 50% of the target bacteria; ^b^pool-D has high STREP14 killing titer that exceeded the upper limit of the assay.

The ELISA and OPA data suggest that adding saponin adjuvant VSA-1 to PCV13 is a viable way to boost antibody responses and increase opsonic antibodies induced by PCV13. Both VSA-1 and QS-21 boosted IgG and OPA titers against the tested serotypes, including serotypes 3, 14, and 19A that are involved in most PCV13 breakthroughs, especially serotype 3. VSA-1 and QS-21 are known for stimulating antigen-specific humoral and cellular immunity. They can potentially enhance the serotype-specific immune memory and help to reduce number of immunizations of PCV13 while maintaining a high level of protection. The adjuvants’ capability in stimulating cellular immune responses can also help to overcome immunosenescence and improve efficacy of glycoconjugate pneumococcal vaccine in elderly (44), which is important given that PCV13 is 75% effective against IPD in adults older than 65 years. Since VSA-1 is much more accessible and of lower toxicity than QS-21, it can be a practical saponin immunostimulant to be included in a new glycoconjugate pneumococcal vaccine formulation. However, development of a new adjuvant requires studies in different animal species and in human. For future development, we plan to perform additional studies in different animal models and optimize the adjuvant doses and immunization regimens.

## Data availability statement

The original contributions presented in the study are included in the article. Further inquiries can be directed to the corresponding authors.

## Ethics statement

The animal study was reviewed and approved by The Institutional Animal Care and Use Committee of the University of Alabama at Birmingham.

## Author contributions

MN and PW designed the study and experiments and wrote the paper. HK and DB conducted the synthesis and immunizations. JY and HK conducted ELISA analysis and JY conducted OPA analysis. All authors read and approved the manuscript. All authors contributed to the article and approved the submitted version.

## References

[1] Theilacker C Fletcher MA Jodar L Gessner BD. PCV13 vaccination of adults against pneumococcal disease: What we have learned from the community-acquired pneumonia immunization trial in adults (CAPiTA). Microorganisms (2022) 10(1):127. doi: 10.3390/microorganisms10010127 PMC8778913 35056576

[2] Lee LH Gu XX Nahm MH. Towards new broader spectrum pneumococcal vaccines: The future of pneumococcal disease prevention. Vaccines (Basel). (2014) 2(1):112–28. doi: 10.3390/vaccines2010112 PMC4494192 26344470

[3] Dransfield MT Harnden S Burton RL Albert RK Bailey WC Casaburi R. Long-term comparative immunogenicity of protein conjugate and free polysaccharide pneumococcal vaccines in chronic obstructive pulmonary disease. Clin Infect Dis (2012) 55(5):e35–44. doi: 10.1093/cid/cis513 PMC3491850 22652582

[4] Kim YK LaFon D Nahm MH. Indirect effects of pneumococcal conjugate vaccines in national immunization programs for children on adult pneumococcal disease. Infect Chemother (2016) 48(4):257–66. doi: 10.3947/ic.2016.48.4.257 PMC5204004 28032483

[5] Jackson LA El Sahly HM George S Winokur P Edwards K Brady RC. Randomized clinical trial of a single versus a double dose of 13-valent pneumococcal conjugate vaccine in adults 55 through 74years of age previously vaccinated with 23-valent *pneumococcal* polysaccharide vaccine. Vaccine (2018) 36(5):606–14. doi: 10.1016/j.vaccine.2017.12.061 PMC5777223 29279281

[6] Gruber WC Scott DA Emini EA. Development and clinical evaluation of prevnar 13, a 13-valent pneumocococcal CRM197 conjugate vaccine. Ann NY Acad Sci (2012) 1263:15–26. doi: 10.1111/j.1749-6632.2012.06673.x 22830997

[7] Masomian M Ahmad Z Gew LT Poh CL. Development of next generation *streptococcus pneumoniae* vaccines conferring broad protection. Vaccines (Basel). (2020) 8(1):132. doi: 10.3390/vaccines8010132 PMC7157650 32192117

[8] Mokaddas E Syed S Albert MJ. The 13-valent pneumococcal conjugate vaccine (PCV13) does not appear to provide much protection on combined invasive disease due to the six PCV13 non-PCV7 serotypes 1, 3, 5, 6A, 7F, and 19A in Kuwait during 2010-2019. Hum Vaccin Immunother. (2021) 17(11):4661–6. doi: 10.1080/21645515.2021.1968216 PMC8828145 34435932

[9] Backhaus E Berg S Andersson R Ockborn G Malmström P Dahl M. Epidemiology of invasive pneumococcal infections: manifestations, incidence, and case fatality rate correlated to age, gender, and risk factors. BMC Infect Dis (2016) 16:367. doi: 10.1186/s12879-016-1648-2 27487784 PMC4972955

[10] Andrews NJ Waight PA Burbidge P Pearce E Roalfe L Zancolli M. Serotype-specific effectiveness and correlates of protection for the 13-valent *pneumococcal conjugate* vaccine: A postlicensure indirect cohort study. Lancet Infect Dis (2014) 14:839–46. doi: 10.1016/S1473-3099(14)70822-9 25042756

[11] Silva-Costa C Gomes-Silva J Pinho MD Friães A Ramirez M Melo-Cristino J. Continued vaccine breakthrough cases of serotype 3 complicated pneumonia in vaccinated children, Portugal (2016–2019). Microbiol Spectr (2022) 10(4):e01077-22. doi: 10.1128/spectrum.01077-22 PMC9431508 35862941

[12] Dominguez Â Ciruela P Hernandez S Garcia-Garcia JJ Soldevila N Izquierdo C. Effectiveness of the 13-valent pneumococcal conjugate vaccine in preventing invasive pneumococcal disease in children aged 7-59 months. A matched case-control study. PloS One (2017) 12(8):e0183191. doi: 10.1371/journal.pone.0183191 28806737 PMC5555701

[13] Horacio AN Silva-Costa C Lopes JP Ramirez M Melo-Cristino J. Serotype 3 remains the leading cause of invasive pneumococcal disease in adults in portugal (2012–2014) despite continued reductions in other 13-valent conjugate vaccine serotypes. Front Microbiol (2016) 7:1616. doi: 10.3389/fmicb.2016.01616 27790208 PMC5064670

[14] Ludwig G Garcia-Garcia S Lanaspa M Ciruela P Esteva C Fernandez de Sevilla M. Serotype and clonal distribution dynamics of invasive pneumococcal strains after PCV13 introduction (2011-2016): Surveillance data from 23 sites in Catalonia, Spain. PloS One (2020) 15(2):e0228612. doi: 10.1371/journal.pone.0228612 32027715 PMC7004304

[15] Cohen R Levy C Ouldali N Goldrey M Bechet S Bonacorsi S. Invasive disease potential of pneumococcal serotypes in children after PCV13 implementation. Clin Infect Dis (2021) 72(8):1453–6. doi: 10.1093/cid/ciaa917 32804200

[16] Yoon JG Jang AY Kim MJ Seo YB Lee J Choi YH. Persistent serotype 3 and 19A invasive pneumococcal diseases in adults in vaccine era: Serotype-dependent difference in ceftriaxone susceptibility. Vaccine (2022) 40(15)2258–2265. doi: 10.1016/j.vaccine.2022.03.004 35282927

[17] Mungall BA Hoet B Guevara JN Soumahoro L. A systematic review of invasive pneumococcal disease vaccine failures and breakthrough with higher-valency pneumococcal conjugate vaccines in children. Expert Rev Vaccines (2022) 21(2):201–14. doi: 10.1080/14760584.2022.2012455 34882050

[18] Leroux-Roels I Devaster JM Leroux-Roels G Verlant V Henckaerts I Moris P. Adjuvant system AS02V enhances humoral and cellular immune responses to pneumococcal protein PhtD vaccine in healthy young and older adults: randomised, controlled trials. Vaccine (2015) 33(4):577–84. doi: 10.1016/j.vaccine.2013.10.052 24176494

[19] Bonam SR Partidos CD Halmuthur SKM Muller S. An overview of novel adjuvants designed for improving vaccine efficacy. Trends Pharmacol Sci (2017) 38(9):771–93. doi: 10.1016/j.tips.2017.06.002 28668223

[20] Di Pasquale A Preiss S Tavares Da Silva F Garcon N. Vaccine adjuvants: from 1920 to 2015 and beyond. Vaccines (Basel). (2015) 3(2):320–43. doi: 10.3390/vaccines3020320 PMC4494348 26343190

[21] O’Hagan DT Friedland LR Hanon E Didierlaurent AM. Towards an evidence based approach for the development of adjuvanted vaccines. Curr Opin Immunol (2017) 47:93–102. doi: 10.1016/j.coi.2017.07.010 28755542

[22] Garçon N Leroux-Roels G Cheng W-F. Vaccine adjuvants. In: Garçon N Stern PL Cunningham AL, editors. Understanding modern vaccines perspectives in vaccinology. Amsterdam: Elsevier (2011). p. 89–113.

[23] Giudice GD Rappuoli R Didierlaurent AM. Correlates of adjuvanticity: A review on adjuvants in licensed vaccines. Semin Immunol (2018) 2018:14–21. doi: 10.1016/j.smim.2018.05.001 29801750

[24] Shi S Zhu H Xia X Liang Z Ma X Sun B. Vaccine adjuvants: Understanding the structure and mechanism of adjuvanticity. Vaccine (2019) 37:3167–78. doi: 10.1016/j.vaccine.2019.04.055 31047671

[25] Didierlaurent AM Laupeze B Di Pasquale A Hergli N Collignon C Garcon N. Adjuvant system AS01: helping to overcome the challenges of modern vaccines. Expert Rev Vaccines (2017) 16(1):55–63. doi: 10.1080/14760584.2016.1213632 27448771

[26] James SF Chahine EB Sucher AJ Hanna C. Shingrix: the new adjuvanted recombinant herpes zoster vaccine. Ann Pharmacother (2018) 52(7):673–80. doi: 10.1177/1060028018758431 29457489

[27] Garcon N Van Mechelen M. Recent clinical experience with vaccines using MPL- and QS-21-containing adjuvant systems. Expert Rev Vaccines (2011) 10(4):471–86. doi: 10.1586/erv.11.29 21506645

[28] Cunningham AL Lal H Kovac M Chlibek R Hwang S-J Díez-Domingo J. Efficacy of the herpes zoster subunit vaccine in adults 70 years of age or older. New Engl J Med (2016) 375(11):1019–32. doi: 10.1056/NEJMoa1603800 27626517

[29] Lacaille-Dubois MA. Updated insights into the mechanism of action and clinical profile of the immunoadjuvant QS-21: A review. Phytomedicine (2019) 60:152905. doi: 10.1016/j.phymed.2019.152905 31182297 PMC7127804

[30] Ragupathi G Gardner JR Livingston PO Gin DY. Natural and synthetic saponin adjuvant QS-21 for vaccines against cancer. Expert Rev Vaccines (2011) 10:463–70. doi: 10.1586/erv.11.18 PMC3658151 21506644

[31] Martin RS Briones R. Industrial uses and sustainable supply of quillaja saponaria (Rosaceae) saponins. Economic Botany. (1999) 53(3):302–11. doi: 10.1007/BF02866642

[32] Wang P Dai Q Thogaripally P Zhang P Michalek SM. Synthesis of QS-21-based immunoadjuvants. J Org Chem (2013) 78:11525–34. doi: 10.1021/jo402118j PMC3937867 24147602

[33] Wang P Devalankar DA Dai Q Zhang P Michalek SM. Synthesis and evaluation of QS-21-based immunoadjuvants with a terminal-functionalized side chain incorporated in the west wing trisaccharide. J Org Chem (2016) 81:9560–6. doi: 10.1021/acs.joc.6b00922 PMC6488304 27709937

[34] Wang P Ding X Kim H SŠkalamera Đ Michalek SM Zhang P. Vaccine adjuvants derivatized from momordica saponins I and II. J Med Chem (2019) 62:9976–82. doi: 10.1021/acs.jmedchem.9b01511 PMC6856410 31657920

[35] Wang P Škalamera Đ Sui X Zhang P Michalek SM. Synthesis and evaluation of a QS-17/18-based vaccine adjuvant. J Med Chem (2019) 62:1669–76. doi: 10.1021/acs.jmedchem.8b01997 PMC6492282 30656932

[36] Wang P SŠkalamera Đ Sui X Zhang P Michalek SM. Synthesis and evaluation of QS-7-based vaccine adjuvants. ACS Infect Dis (2019) 5:974–81. doi: 10.1021/acsinfecdis.9b00039 PMC6848976 30920199

[37] Morefield GL. A rational, systematic approach for the development of vaccine formulations. AAPS J (2011) 13(2):191–200. doi: 10.1208/s12248-011-9261-1 21347616 PMC3085699

[38] Wernette CM Frasch CE Madore D Carlone G Goldblatt D Plikaytis B. Enzyme-linked immunosorbent assay for quantitation of human antibodies to pneumococcal polysaccharides. Clin Diagn Lab Immunol (2003) 10(4):514–9. doi: 10.1128/cdli.10.4.514-519.2003 PMC164258 12853378

[39] Burton RL Nahm MH. Development and validation of a fourfold multiplexed opsonization assay (MOPA4) for pneumococcal antibodies. Clin Vaccine Immunol (2006) 13(9):1004–9. doi: 10.1128/CVI.00112-06 PMC1563573 16960111

[40] LaFon DC Nahm MH. Measuring quantity and function of pneumococcal antibodies in immunoglobulin products. Transfusion (2018) 58 Suppl 3:3114–20. doi: 10.1111/trf.15015 30536435

[41] Burton RL Nahm MH. Development of a fourfold multiplexed opsonophagocytosis assay for pneumococcal antibodies against additional serotypes and discovery of serological subtypes in streptococcus pneumoniae serotype 20. Clin Vaccine Immunol (2012) 19(6):835–41. doi: 10.1128/CVI.00086-12 PMC3370448 22518015

[42] Song JY Moseley MA Burton RL Nahm MH. Pneumococcal vaccine and opsonic pneumococcal antibody. J Infect Chemother (2013) 19(3):412–25. doi: 10.1007/s10156-013-0601-1 PMC3692352 23657429

[43] LaFon DC Nahm MH. Measuring immune responses to pneumococcal vaccines. J Immunol Methods (2018) 461:37–43. doi: 10.1016/j.jim.2018.08.002 30098317 PMC6169307

[44] Sterrett S Peng BJ Burton RL LaFon DC Westfall AO Singh S. Peripheral CD4 T follicular cells induced by a conjugated pneumococcal vaccine correlate with enhanced opsonophagocytic antibody responses in younger individuals. Vaccine (2020) 38(7):1778–86. doi: 10.1016/j.vaccine.2019.12.023 PMC8040292 31911030

